# Phage Display-Derived Binders Able to Distinguish *Listeria monocytogenes* from Other *Listeria* Species

**DOI:** 10.1371/journal.pone.0074312

**Published:** 2013-09-10

**Authors:** Mary Josephine McIvor, Nitsara Karoonuthaisiri, Ratthaphol Charlermroj, Linda D. Stewart, Christopher T. Elliott, Irene R. Grant

**Affiliations:** 1 Institute for Global Food Security, School of Biological Sciences, Queen’s University, Belfast, Northern Ireland, United Kingdom; 2 Microarray Laboratory, National Center for Genetic Engineering and Biotechnology, Khlong Laung, Pathum Thani, Thailand; University of Malaya, Malaysia

## Abstract

The objective of this study was to produce phage display-derived binders with the ability to distinguish *Listeria monocytogenes* from other *Listeria* spp., which may have potential utility to enhance detection of *Listeria monocytogenes*. To obtain binders with the desired binding specificity a series of surface and solution phage-display biopannings were performed. Initially, three rounds of surface biopanning against gamma-irradiated *L. monocytogenes* serovar 4b cells were performed followed by an additional surface biopanning round against *L. monocytogenes* 4b which included prior subtraction biopanning against gamma-irradiated *L. innocua* cells. In an attempt to further enhance binder specificity for *L. monocytogenes* 4b two rounds of solution biopanning were performed, both rounds included initial subtraction solution biopanning against *L. innocua*. Subsequent evaluations were performed on the phage clones by phage binding ELISA. All phage clones tested from the second round of solution biopanning had higher specificity for *L. monocytogenes* 4b than for *L. innocua* and three other foodborne pathogens (*Salmonella* spp., *Escherichia coli* and *Campylobacter jejuni*). Further evaluation with five other *Listeria* spp. revealed that one phage clone in particular, expressing peptide GRIADLPPLKPN, was highly specific for *L. monocytogenes* with at least 43-fold more binding capability to *L. monocytogenes* 4b than to any other *Listeria* sp. This proof-of-principle study demonstrates how a combination of surface, solution and subtractive biopanning was used to maximise binder specificity. *L. monocytogenes*-specific binders were obtained which could have potential application in novel detection tests for *L. monocytogenes*, benefiting both the food and medical industries.

## Introduction

*Listeria monocytogenes* is one of six species within the *Listeria* genus, with the other six identified as *L**. grayi* (subsp. *grayi* and subsp. *murrayi*)*, L. innocua, **L**. ivanovii**, **L**. seeligeri*, and *L**. welshimeri* [1,2]. They are Gram positive, facultative anaerobic, non-spore-forming, rod-shaped bacteria 0.5 µm in width and 1-1.5 µm in length [2]. Although *L. monocytogenes* and *L**. ivanovii* are both deemed pathogenic only *L. monocytogenes* infects both man and animals, with *L**. ivanovii* being an animal pathogen rarely occurring in man [3,4]. *Listeria* spp. are ubiquitous in the environment and found in soil, water, effluents, animal and human intestines and, a wide variety of foods including fresh produce. *L. monocytogenes* possess the ability to grow at refrigeration temperatures (<4 °C) making its presence in food a pertinent public health risk where ready-to-eat, refrigerated foods dominate the convenience food market. Although the incidence rate of *L. monocytogenes* infection is lower than that for other foodborne pathogens such as *Salmonella* and *Campylobacter*, the associated mortality rate is much higher [5]. There are thirteen different *L. monocytogenes* serovars based on variation in the somatic (O) and flagellar (H) antigens, however, at least 95% of the strains isolated from foods and patients are serovars 1/2a, 1/2b, 1/2c, and 4b [6–8]. While these four serovars demonstrate varying pathogenicity, it is serovar 1/2a that is the most frequently isolated from food, with serovar 4b causing the majority of human epidemics [8,9].

The current commercially available tests for *Listeria* spp. are mostly culture techniques which tend to be labour and time intensive. To enhance the detection and identification of *L. monocytogenes* more rapid methods are urgently needed to meet current demands for food safety testing. A number of rapid methods have been developed, for example immunological techniques such as biosensor [10,11], enzyme-linked immunosorbent assay (ELISA) [12] and antibody array [13,14] and, genotypic techniques such as real-time polymerase chain reaction (RT-PCR) [15–17], deoxyribonucleic acid (DNA) microarray [18] and loop-mediated isothermal amplification (LAMP) methods [19,20]. *Listeria* spp. are closely related both morphologically and biochemically making it difficult to distinguish *L. monocytogenes* from other *Listeria* spp. Although genotypic techniques that rely on detecting unique DNA sequences between species are available, such techniques can be time-consuming requiring additional sample extraction steps. Culture combined with immunological techniques are used more routinely, however, generating antibodies with the desired specificity and sensitivity for *L. monocytogenes* detection presents the challenge of identifying cell surface epitopes which are specific.

Successful rapid diagnostic techniques require a specific and high affinitybinder, usually an antibody. Development of animal derived antibodies is not an easy task and they are expensive to produce. In addition efforts are ongoing to reduce, refine and replace the use of animals in scientific research in accordance with EU legislation, Directive 2010/63/EU [21]. Phage display biopanning is a technique extending from the invention of phage display technology in 1985 [22] and offers an alternative means of generating specific affinity ligands. This technique has been used previously in an attempt to generate alternative binders to *L. monocytogenes*; Paoli et al. [5,23] and Nanduri et al. [24] produced phage display-derived antibody fragments to *L. monocytogenes*, while Carnazza et al. [25] described the production of phage display-derived peptide binders to *L. monocytogenes*. However, Carnazza et al. presented no data on cross-reactivity of their binders with other *Listeria* spp. or relevant foodborne bacteria to support the specificity claim.

This study aimed to use phage display biopanning to produce *L. monocytogenes*-specific binders with the ability to distinguish pathogenic *L. monocytogenes* from other *Listeria* species. The biopanning regime employed subtraction biopanning against *L. innocua*, the strain genetically closest to *L. monocytogenes* [26], in the hope that this would increase the probability of identifying *L. monocytogenes*-specific binders. The ultimate goal was to determine the potential utility of the phage display-derived *L. monocytogenes*-specific binders in a rapid diagnostic assay to enhance the detection and identification of this important foodborne pathogen.

## Materials and Methods

### Preparation of target antigen and biopanning plates

Target antigen for positive surface biopanning was *L. monocytogenes* NCTC 4885 (serovar 4b) and for subtraction surface biopanning was *L. innocua* NCTC 11288 (Table 1). The bacteria were maintained on Cryobeads (Pro-Lab, Wirral, UK) at -80 ^°^C. Each culture was prepared by the inoculation of nutrient broth (CM0001, Oxoid Limited, Hampshire, UK) with a single Cryobead followed by incubation at 30 ^°^C for 48 hours. Each culture was standardized to 2x10^9^ cfu ml^-1^ in phosphate buffered saline pH 7.4 (PBS) and subjected to a 10 kGy dose of gamma radiation (using a Gammabeam 650 irradiator located at Agri-Food and Biosciences Institute for Northern Ireland, Belfast, UK). Once irradiated the cultures underwent a 10-fold concentration with final resuspension in 0.1 M sodium hydrogen carbonate pH 8.6 (coating buffer). Four 60 mm Petri dishes (Sarstedt, Leicester, UK) were coated with 1.5 ml of *L. monocytogenes* (2x10^10^ cfu ml^-1^) and one Petri dish with 1.5 ml of *L. innocua* (2x10^10^ cfu ml^-1^) and incubated with agitation at 4 ^°^C in a humidified container until required.

**Table 1:** Bacterial strains employed in this study.

Bacterium	Serogroup	Source
*Listeria monocytogenes*	4b	NCTC 4885
*Listeria monocytogenes*	1/2a	Environmental swab^a^
*Listeria monocytogenes*	1/2b	Environmental swab^a^
*Listeria monocytogenes*	1/2c	Environmental swab^a^
*Listeria innocua*	-	NCTC 11288
*Listeria* *grayi* subsp. *grayi*	-	ATCC 19120^b^
*Listeria* *grayi* subsp. *murrayi*	-	NCTC 10812 ^b^
*Listeria* *ivanovii*	-	NCTC 11846 ^b^
*Listeria* *seelgeri*	-	NCTC 11856 ^b^
*Listeria* *welshimeri*	-	Environmental swab^a^
*Salmonella* Enteriditis	9,12:g,m:7	NCTC 6676
*Salmonella* Typhimurium	4,12:i:2	Pig carcass swab^c^
*Salmonella* Dublin	9,12:g,p	Pork^c^
*Salmonella* Infantis	6,7:r:5	Raw chicken^c^
*Salmonella* Senftenberg	3,19:g,s,t	Animal feed^c^
*Salmonella* Hadar	6,8:z10:e,n,x	QA sample- LGC^d^
*Salmonella* Mbandaka	6,7:z10:e,n,z15	Hygiene swab^c^
*Salmonella* Virchow	6,7:r:2	NCTC 5742
*Escherichia coli* K12	-	NCTC 10538
*Campylobacter jejuni*	-	NCTC 11351

^a^Originally isolated from environmental swabs taken at food processing facilities and serotyped by Teagasc Food Research Centre, Moorepark, Republic of Ireland. Kindly provided by Dr Kieran Jordan

^b^Kindly provided by Mr Mark Linton, Agri-Food and Biosciences Institute for Northern Ireland, Belfast, UK

^c^Originally isolated and serotyped by *Salmonella* Reference Laboratory, Agri-Food and Biosciences Institute for Northern Ireland, Belfast, UK. Kindly provided by Dr Robert Madden

^d^Laboratory of the Government Chemist, Middlesex, UK

Other bacteria used (Table 1) were maintained on Cryobeads and were cultured and irradiated in the same manner as described above, *Salmonella* serovars and *Escherichia coli* were incubated at 37 ^°^C overnight, and *Campylobacter jejuni* incubated at 42 ^°^C for 48 hours. The *Salmonella* spp. cocktail consisted of an equal ratio mix of the eight *Salmonella* serovars most commonly associated with foodborne outbreaks in Europe [27]. The bacteria listed in Table 1 were also prepared in a heat-killed (heat treated to 80 ^°^C for 15 minutes) form.

### Production of phage clone binders to L. monocytogenes by phage display biopanning

#### Surface biopanning

**Figure 1:**
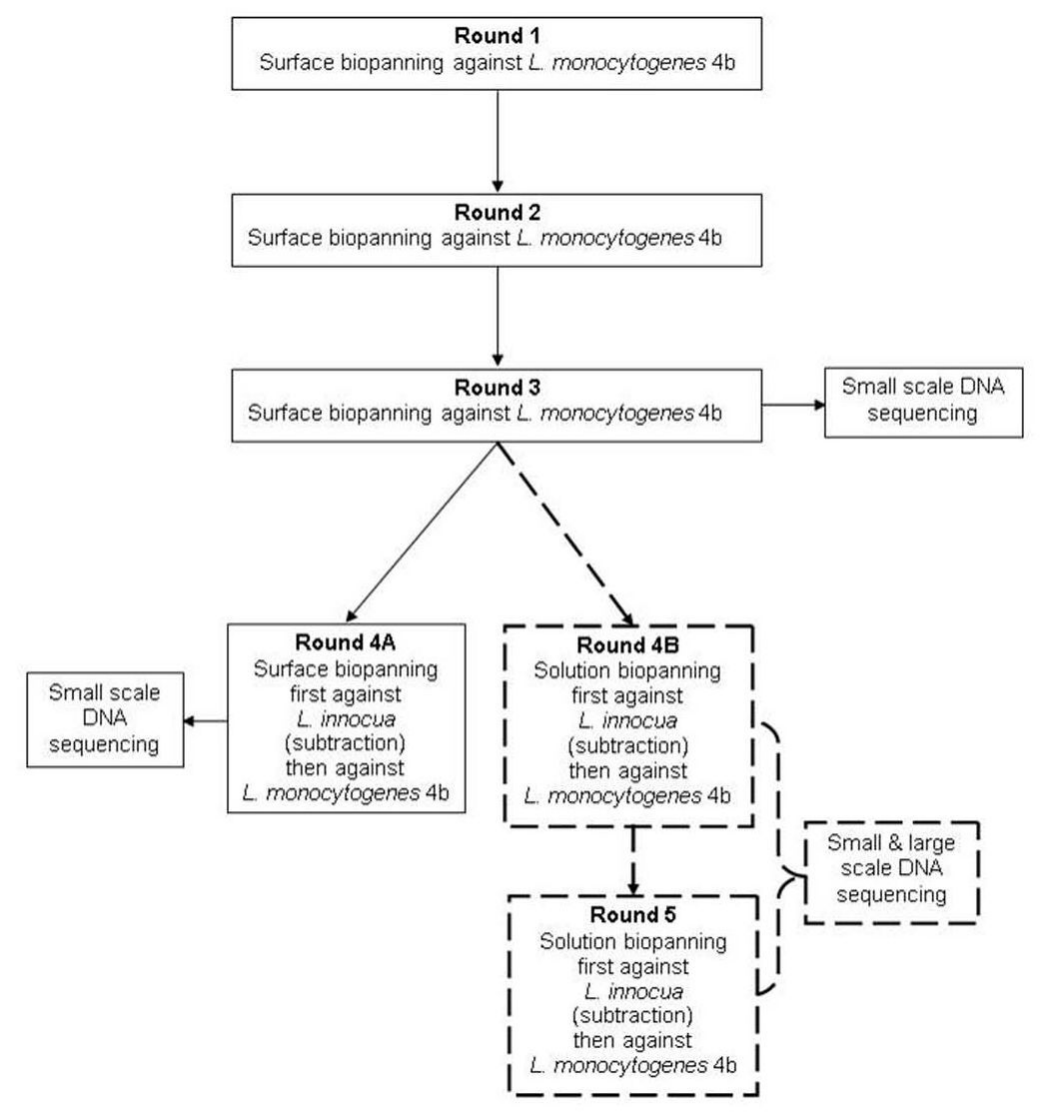
Schematic of phage display biopanning regime adopted in order to generate *L. monocytogenes-*specific binders.

Four surface biopannings (Figure 1) were performed using a phage display peptide library [Ph.D.-12, New England Biolabs (NEB) Hertfordshire, UK] according to the kit instructions. The first three rounds were performed against *L. monocytogenes* to select for phages binding to *L. monocytogenes*. The fourth included an initial subtraction biopanning step performed against *L. innocua* and using the supernatant derived from that for the subsequent biopanning against *L. monocytogenes*.

#### Solution biopanning

Two solution biopannings (Figure 1) were performed following the completion of the third surface biopanning round and the protocol based on a combination of the surface biopanning protocol in the NEB kit instructions and the solution biopanning protocol employed by Paoli et al. [5]. Each round included one solution biopanning against viable *L. innocua* and one against viable *L. monocytogenes* 4b. The cultures were prepared and standardized to 2x10^9^ cfu ml^-1^ in the same manner as described above and the bacterial cells washed twice in tris buffered saline pH 7.5 (TBS), before final resuspension with a blocking solution [TBS-1 % bovine serum albumin (BSA)] to the original volume. Four sterile 1.5 ml Eppendorf tubes (Eppendorf 1, 2, 3 and 4) were blocked with 1 ml of blocking solution and mixed for 1 hour at 20 ^°^C on a rotator mixer (Stuart Scientific, UK) and prior to their use the blocking solution was discarded. *L. innocua* (750 µl, 2 x 10^9^ cfu ml^-1^) was added to Eppendorf 1 and rotated (1 hour at 20 ^°^C) followed by 100 µl of amplified phage (containing 10^12^ phage particles) from surface biopanning round 3 with rotation as above. While this incubation was underway 750 μl of *L. monocytogenes* (2x10^9^ cfu ml^-1^) was added to Eppendorf 2 with rotation as above. Eppendorf 1 was centrifuged (10,000 g for 2 minutes at 20 ^°^C), the supernatant collected and added to Eppendorf 2 with rotation as above. Eppendorf 2 was centrifuged and the pellet of phages bound to *L. monocytogenes* washed five times with TBS-0.5 % Tween 20 followed by five times with TBS. For the final wash step the resuspended pellet was transferred to Eppendorf 3 (to eliminate the possibility of eluting phage that may have bound to Eppendorf 2) and the pellet resuspended in 500 μl of elution buffer [0.2 M glycine-hydrochloric acid (HCl) pH 2.2] with rotation for 20 minutes at 20 ^°^C. Eppendorf 3 was centrifuged, the supernatant containing phages bound to *L. monocytogenes* collected, added to Eppendorf 4 and neutralized with 75 μl of 1 M Tris-HCl pH 9.1. This unamplified phage from solution biopanning round 4B was tittered and amplified. This solution biopanning protocol was repeated one additional time (round 5).

### DNA sequencing and phage binding ELISA

After rounds 3 and 4A surface biopanning, and rounds 4B and 5 solution biopanning, eighteen phage clones were randomly selected for small scale DNA sequencing by DNA Sequencing and Services, Dundee, UK. In addition, after rounds 4B and 5 solution biopanning, a larger number of phage clones (n=192) were selected for larger scale DNA sequencing by Macrogen, Korea. All corresponding 12-mer peptide sequences were deciphered using FinchTV software (http://www.geospiza.com/Products/finchtv.shtml) and ExPASy translate tool (http://web.expasy.org/translate/) and the consensus between peptide sequences was assessed using online ClustalW2 software (http://www.ebi.ac.uk/Tools/msa/clustalw2/). The SAROTUP tool [28] was used to ensure deciphered peptide sequences were not previously recognised target-unrelated peptide sequences [29]. Unique peptide sequences were screened for their ability to bind to *L. monocytogenes* 4b and four other bacteria, *L. innocua, **Salmonella* spp. cocktail, *E. coli* and *C. jejuni* (Table 1) at 1x10^8^ cfu ml^-1^. The phage binding ELISA protocol (antigen-coated format) was carried out as described in NEB phage display kit instructions. Stock cultures of *L. monocytogenes* 4b*, L. innocua, **Salmonella* spp. cocktail and *C. jejuni* stock had been gamma irradiated whereas *E. coli* stock had been heat-killed.

On the basis of the phage binding ELISA results the 12-mer peptides expressed by eight phage clones showing greatest capability to bind *L. monocytogenes* 4b were chemically synthesised (GL Biochem, Shanghai, China) at > 85% purity (Table 2). The synthesised peptides were subsequently evaluated for magnetic separation (MS) and/or sandwich ELISA applications (Table 3).

**Table 2:** Summary of biopanning outcomes.

Biopanning Round	Type of biopanning	No. of phage clones sequenced	No. of consensus^a^ peptide sequences	Consensus peptide sequence(s) (no. of phage clones)	No. of phage clones tested by phage binding ELISA	No. of phage clones able to bind *L. monocytogenes* 4b
3	Surface	18	1	MGTTHLPYQFSL (3)	13	1
4A	Surface	18	2	KQATFDDYPVAH (7)	7	7
				MGTTHLPYQFSL (2)		
4B	Solution	18	2	KQATFDDYPVAH (3)	0^b^	-
				KLHISKDHIYPT(2)		
	Solution	192	12	see Table S1	0^b^	-
5	Solution	18	1	GPLATLHLPHKT (2)	12	11
	Solution	192	29	see Table S2	12	12

^a^consensus meaning two or more phage clones expressing the same peptide sequence

^b^No phage binding ELISA performed - decision was made to wait until after round 5 to perform further phage binding ELISA

**Table 3:** Information on peptides chemically synthesised and evaluated for specific binding to *L. monocytogenes* 4b by either magnetic separation (MS) or sandwich ELISA.

Biopanning Round	Type of biopanning	Phage clone identifier	Expressed peptide sequence	Synthesised peptide sequence	Tested by MS + plate counts	Tested by sandwich ELISA
3	Surface	LM0315	KQATFDDYPVAH	KQATFDDYPVAH**GGGSC**^a^	✓	×
4A	Surface	LM0411	LCAKTKLHNKTY	LCAKTKLHNKTY**GGGSC**	✓	×
		LM0418	ANPRKVRLQRNK	ANPRKVRLQRNK**GGGSC**	✓	×
		LM0408	RKQNRNKSLPTN	RKQNRNKSLPTN**GGGSC**[**BSA**^b^]	×	✓
		LM0418	ANPRKVRLQRNK	ANPRKVRLQRNK**GGGSC[BSA]**	×	✓
5	Solution	LM020502	GKIWTEPPPPKP	GKIWTEPPPPKP**GGGSC**	✓	×
		LM020507	GVIYDKPA-KLH	GVIYDKPA**GGGSC**	✓	×
		LM020509	GPLATLHLPHKT	GPLATLHLPHKT**GGGSC**	✓	×

^a^GGGSC: 5-amino acid spacer recommended by NEB phage display kit manual

^b^Carrier protein BSA added for detection purposes in sandwich ELISA

### Evaluation of the binding capability of synthetic peptides

#### (a) Magnetic separation

Synthetic peptides (Table 3) were coupled to MyOne™ tosylactivated Dynabeads® (Life Technologies Limited, Paisley, UK) as per the manufacturer’s instructions, using 1 mg of peptide to coat 25 mg of beads (10^11^ beads ml^-1^). Protein concentration was measured at 280 nm for post-coupling supernatants from the preparation of peptide-coated beads to verify that coupling had occurred. A Dynal® BeadRetriever (Life Technologies Limited, Paisley, UK) was used for automated MS. Serial dilutions (10-fold) of *L. monocytogenes* 4b or *L. innocua* prepared in nutrient broth (containing 10^1^–10^7^ cfu ml^-1^) were tested. The MS protocol used peptide-coated beads on duplicate aliquots of two cell concentrations of *L. monocytogenes* and *L. innocua* (3x10^2^ and 3x10^3^ cfu ml^-1^). The protocol was as follows; capture of cells by beads for 30 minutes at very slow speed (1 ml bacterial dilution and 10 μl beads), 10 second release of beads at very fast speed, with two washes for 1 minute in 1 ml PBS with 0.05% Tween 20 (PBST) at very slow speed followed by 10 second release of beads at fast speed with final 2 minutes elution at fast speed into 100 μl of nutrient broth (thus a 10-fold concentration was achieved during MS). Alongside the peptide-coated beads, commercially available antibody-coated beads (anti-*Listeria* Dynabeads®, Life Technologies Limited, Paisley, UK) were included as a positive control. Plate counts were performed pre- and post-MSfor each bead type by spreading 100 µL sample, or an appropriate dilution, onto a nutrient agar plate and incubating at 30 ^°^C for 48 hours. The concentrations of 3x10^2^ and 3x10^3^ cfu ml^-1^ were chosen because a countable number of colonies would result if capture occurred. The protocol was performed in duplicate and mean % capture values for each bead type with each bacterium subsequently calculated.

#### (b) Sandwich ELISA

Preliminary trials established that the optimized blocking and diluting buffers for ELISA were 3% and 1% skimmed milk in PBST, respectively. For specificity studies a Nunc Maxisorp ELISA plate (Nunc, New York, USA) was coated with 100 µl of synthetic peptide-BSA conjugates, LM0408-BSA and LM0418-BSA (Table 3), at 50 µg ml^-1^ in 50 mM sodium carbonate-bicarbonate buffer pH 9.6 with incubation overnight at 4 ^o^C. The plate was washed three times with 300 µl of PBST before adding 300 µl of blocking buffer with incubation for 1 hour at room temperature. The plate was washed as described above before the addition of 100 µl of bacteria (Table 1) at 1x10^8^ cfu ml^-1^ with incubation for 1 hour at 20 ^°^C. Stock cultures of *L. monocytogenes* 4b*, L. innocua, **Salmonella* spp. cocktail and *C. jejuni* stock had been irradiated whereas *E. coli* stock had been heat-killed. The plate was washed as described above before the addition of 100 µl of 4 µg ml^-1^ *Listeria*-specific monoclonal antibody 3C3 (Monoclonal Antibody Production Laboratory, BIOTEC, Thailand) with incubation for 1 hour at room temperature. The plate was washed as described above before goat anti-mouse horseradish peroxidase (final concentration of 0.7 g ml^-1^, P0447, Dako Corporation, Denmark) was added with incubation for 1 hour at room temperature in the dark. The 3,3′,5,5′-tetramethylbenzidine (TMB) substrate (Thermo, Fisher Scientific, USA) was added with incubation for 15 minutes at 37 ^°^C with agitation before the developing reaction was stopped with 2.5 M sulphuric acid. Absorbance was immediately read at OD 450 nm using a plate reader (Tecan Safire2, Switzerland). For sensitivity studies the same ELISA protocol was used with a total of 11 bacterial concentrations (half-log increments from 1x10^3^-1x10^8^ cfu ml^-1^). The intensities were used to fit on a dose–response curve with equation [14,30]:


$${\text{Y = A + B / (1 + 10}}^{\text{C-X}}\text{)}$$

Y is the intensity when detecting bacteria at concentration X, while A, B, and C are constants obtained from the curve fitting. The limit of detection (LOD) was calculated using the intensity values greater than twice the background [31,32].

### Evaluation of additional selected phage clones

Seventeen phage clones were selected from throughout the biopanning series for more extensive evaluation by phage binding ELISA (Table 4). Eight of the 17 had their equivalent synthetic peptides previously evaluated by MS and sandwich ELISA (Table 3) and the other nine were from solution panning round 5. For the specificity study the phage clones were used at 1x10^11^ pfu ml^-1^ in PBS-0.1 % Tween 20 and the phage binding ELISA protocol was essentially as described above. However, for these experiments viable bacteria (*L. monocytogenes* 4b*, L. innocua, **Salmonella* spp. cocktail, *E. coli* and *C. jejuni*) were employed, prepared and standardized as described earlier. Each phage clone evaluation was performed in duplicate. Mean OD signals for each phage clone were calculated and the relative binding capability, expressed as ‘fold difference’ between the normalized OD signal for *L. monocytogenes* 4b divided by the normalized OD signal for the other bacteria determined. Based on the fold difference results the four best *L. monocytogenes* 4b-binding phage clones (LM020507, LM020509, LM0205P02B02 and LM0205P01H01) were further evaluated with three additional serovars of *L. monocytogenes* (1/2a, 1/2b, 1/2c) and six other *Listeria* spp., *L**. grayi* subsp. *grayi**, L. innocua, **L**. ivanovii**, **L**. grayi* subsp. *murrayi**, **L**. seelgeri* and *L**. welshimeri*, in the same manner as described above.

**Table 4:** Information on phage clones from biopanning rounds 3, 4A and 5 chosen for further evaluation in terms of specificity for *L. monocytogenes* 4b by phage binding ELISA.

Biopanning Round	Type of biopanning	Phage clone identifier	Expressed sequence	Ability to distinguish *L. monocytogenes* 4b from *L. innocua*
3	Surface	LM0315 ^a^	KQATFDDYPVAH	×
4A	Surface	LM0408 ^a^	ANPRKVRLQRNK	×
		LM0409 ^a^	KLHISKDHIYPT	×
		LM0411 ^a^	LCAKTKLHNKTY	×
		LM0418 ^a^	ANPRKVRLQRNK	×
5	Solution	LM020502 ^a^	GKIWTEPPPPKP	✓
		LM020503 ^a^	GPIFQSQLKSQT	✓
		LM020504 ^a^	GPLVDLGPGDLR	✓
		LM020505 ^a^	GVIYSKPNSVQL	✓
		LM020507 ^a^	GVIYDKPA-KLH	✓
		LM020509 ^a^	GPLATLHLPHKT	✓
		LM0205P01B07 ^b^	GNLFASPQKMH	✓
		LM0205P02A10 ^b^	GPLISTPRHMNI	✓
		LM0205P01D05 ^b^	GAMHLPWHMGT	✓
		LM0205P02B02 ^b^	GPIRDIGPVMDH	✓
		LM0205P01H01 ^b^	GRIADLPPLKPN	✓
		LM0205P02D06 ^b^	GPIYSTQHMKTS	✓

^a^Identified via small scale DNA sequencing

^b^Identified via large scale DNA sequencing

For the sensitivity experiment the same four phage clones were evaluated in the same manner as for the specificity experiment with serial concentrations (10^3^-10^8^ cfu ml^-1^) of viable *L. monocytogenes* 4b and two phage concentrations (1x10^11^ and 1x10^12^ pfu ml^-1^). The experiment was performed in duplicate and LOD values for each phage clone were calculated as described above.

## Results

### Biopanning outcomes

**Figure 2:**
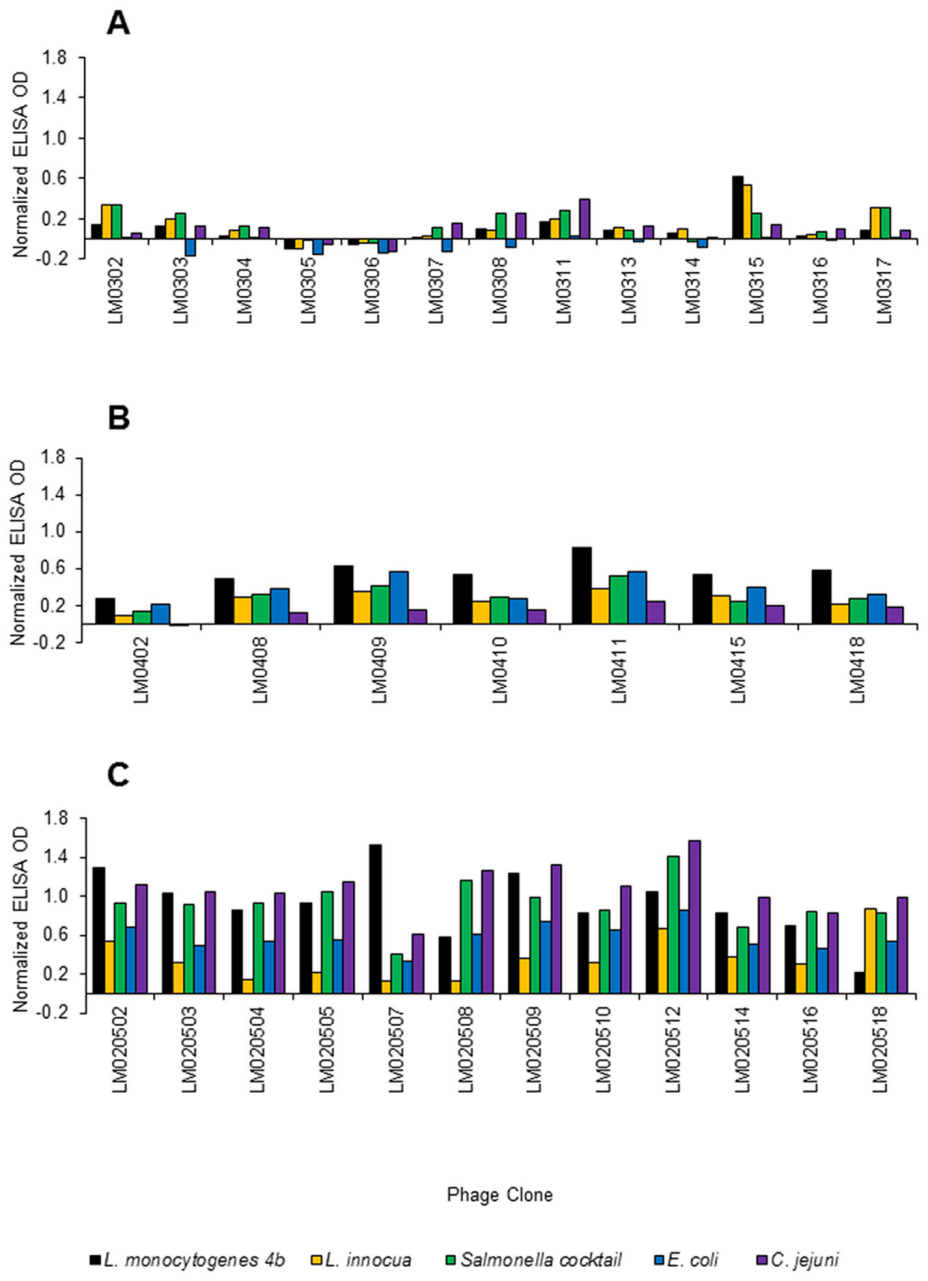
Phage binding ELISA specificity study for phage clones obtained at different stages of biopanning. (A) after surface biopanning round 3 (B) after surface biopanning round 4A and (C) after solution biopanning round 5. Each experiment performed once only due to limited amount of phage clone stock available for testing. Normalized data equates to relative OD signal after subtraction of background OD signal.

The results of biopanning are summarised in Table 2. DNA sequences were obtained for 17 of the 18 phage clones from round 3, with three phage clones having the same peptide sequence, MGTTHLPYQFSL. Thirteen phage clones were screened by phage binding ELISA for their ability to bind *L. monocytogenes* 4b and four other non-target bacteria. Only one phage clone LM0315, expressing peptide KQATFDDYPVAH, exhibited higher binding to *L. monocytogenes* 4b and *L. innocua* than to the other bacteria tested (Figure 2A).

After round 4A, DNA sequences were obtained for 17 of the 18 phage clones, with seven phage clones having the same peptide sequence, KQATFDDYPVAH (also found after round 3 of surface biopanning), and another two phage clones expressing the peptide sequence MGTTHLPYQFSL. Seven phage clones were screened by phage binding ELISA for their ability to bind *L. monocytogenes* 4b and four other non-target bacteria. All seven phage clones exhibited higher binding to *L. monocytogenes* than to the non-target bacteria (Figure 2B).

After the first solution biopanning (round 4B) DNA sequences were obtained for 17 of the 18 phage clones by small scale DNA sequencing, with three phage clones expressing the same peptide sequence, KQATFDDYPVAH (also found in rounds 3 and 4 of surface biopanning), and another two phage clones expressing the same peptide sequence of KLHISKDHIYPT.

After solution panning round 5 DNA sequences were obtained for 15 of the 18 phage clones by small scale DNA sequencing, with two phage clones expressing the same peptide sequence, GPLATLHLPHKT. Twelve phage clones were screened by phage binding ELISA for their ability to bind *L. monocytogenes* 4b and four other non-target bacteria. Except for LM020518, all phage clones exhibited higher binding to *L. monocytogenes* 4b than to *L. innocua* (Figure 2C), two of which, LM020502 and LM020507 expressing peptides GVIYDKPA-KLH and GKIWTEPPPPKP, respectively, also exhibited higher binding to *L. monocytogenes* 4b than to any other bacterium.

Subsequent larger scale DNA sequencing of 192 phage clones randomly selected after rounds 4B and 5 demonstrated more consensus amongst the peptide sequences expressed (Tables S1-S3). Multiple sequence alignment of peptide sequences is shown in Figures S1 and S2. The peptides GAMHLPWHMGTL, GNLFASPQKMHR, GPIRDIGPVMDH, GPIYSTQHMKTS, GPLISTPRHMNI and GRIADLPPLKPN were found amongst round 5 phage clones but not amongst phage clones from earlier surface biopanning rounds.

### Evaluation of the binding capability of synthetic peptides by:

#### (a) Magnetic separation

Protein concentration measurements for post-coupling supernatants demonstrated that synthetic peptides had coupled to the beads to varying degrees. However, % capture values for *L. monocytogenes* 4b of the peptide-coated beads were indicative of non-specific capture (< 5% capture). This was in stark contrast to the positive control, commercial antibody-coated anti-*Listeria* Dynabeads® which achieved close to 100% capture of both *L. monocytogenes* 4b and *L. innocua*.

#### (b) Sandwich ELISA

Specificity studies with peptide-BSA conjugates, LM0408-BSA and LM0418-BSA, demonstrated that both peptides exhibited binding to *L. innocua* and *L. monocytogenes* 4b and low binding to the three other test bacteria (data not shown). Sensitivity studies for both peptide-BSA conjugates with *L. monocytogenes* 4b demonstrated that LM0418-BSA was more sensitive for *L. monocytogenes* 4b detection than LM0408-BSA, with LODs of 4.8x10^6^ cfu ml^-1^ and 1.5x10^7^ cfu ml^-1^, respectively. Sensitivity studies for both peptide-BSA conjugates with *L. innocua* demonstrated that LM0418-BSA was more sensitive for *L. innocua* than LM0408-BSA, with LODs of 6.5x10^6^ cfu ml^-1^ and 7.9x10^6^ cfu ml^-1^, respectively.

### Evaluation of additional selected phage clones

**Figure 3:**
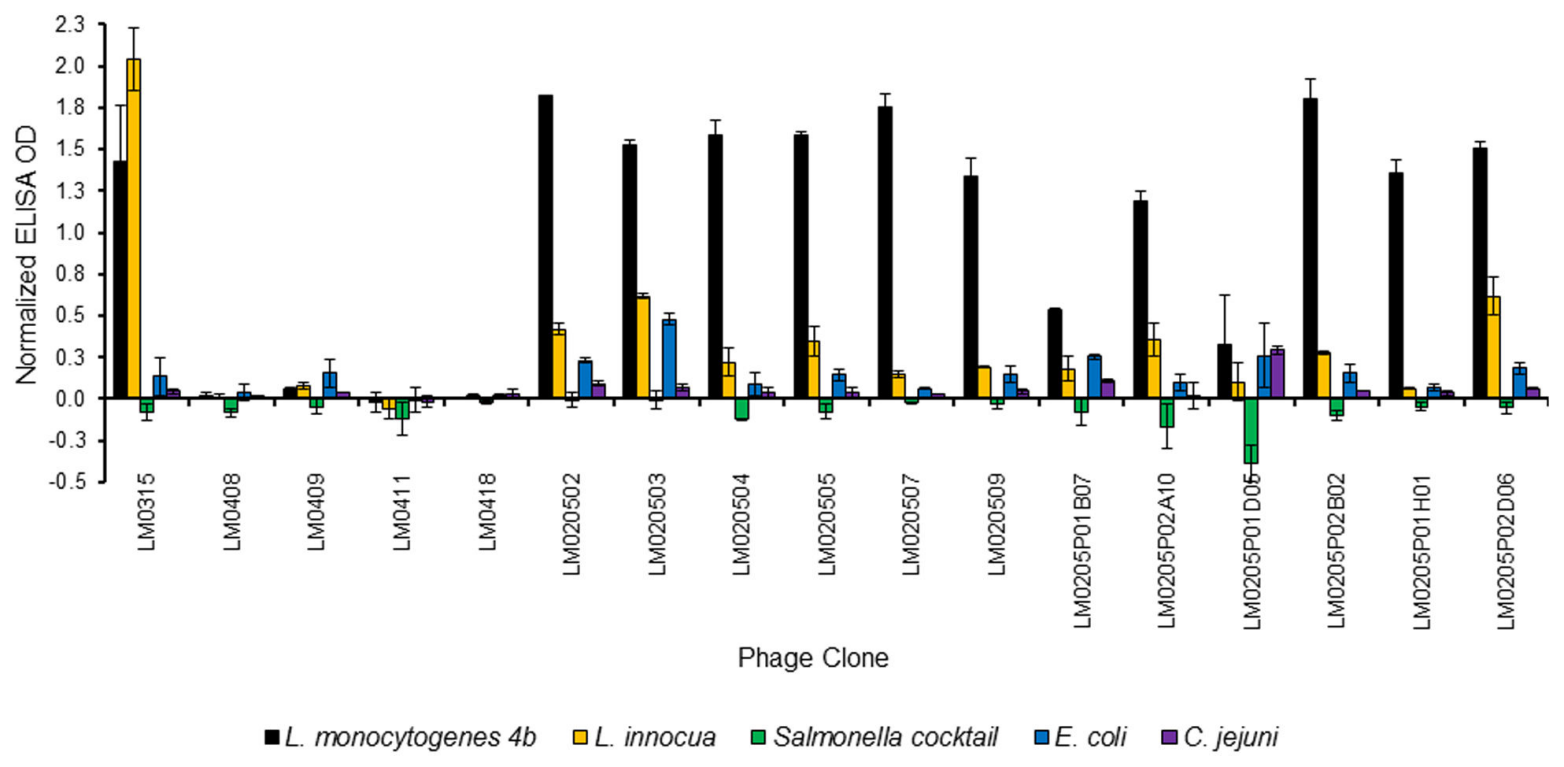
Phage binding ELISA specificity study for 17 selected phage clones from throughout the biopanning series. Normalized data equates to relative OD signal after subtraction of background OD signal. Experiment performed in duplicate (n=2). Error bars represent ± standard deviation of mean.

**Figure 4:**
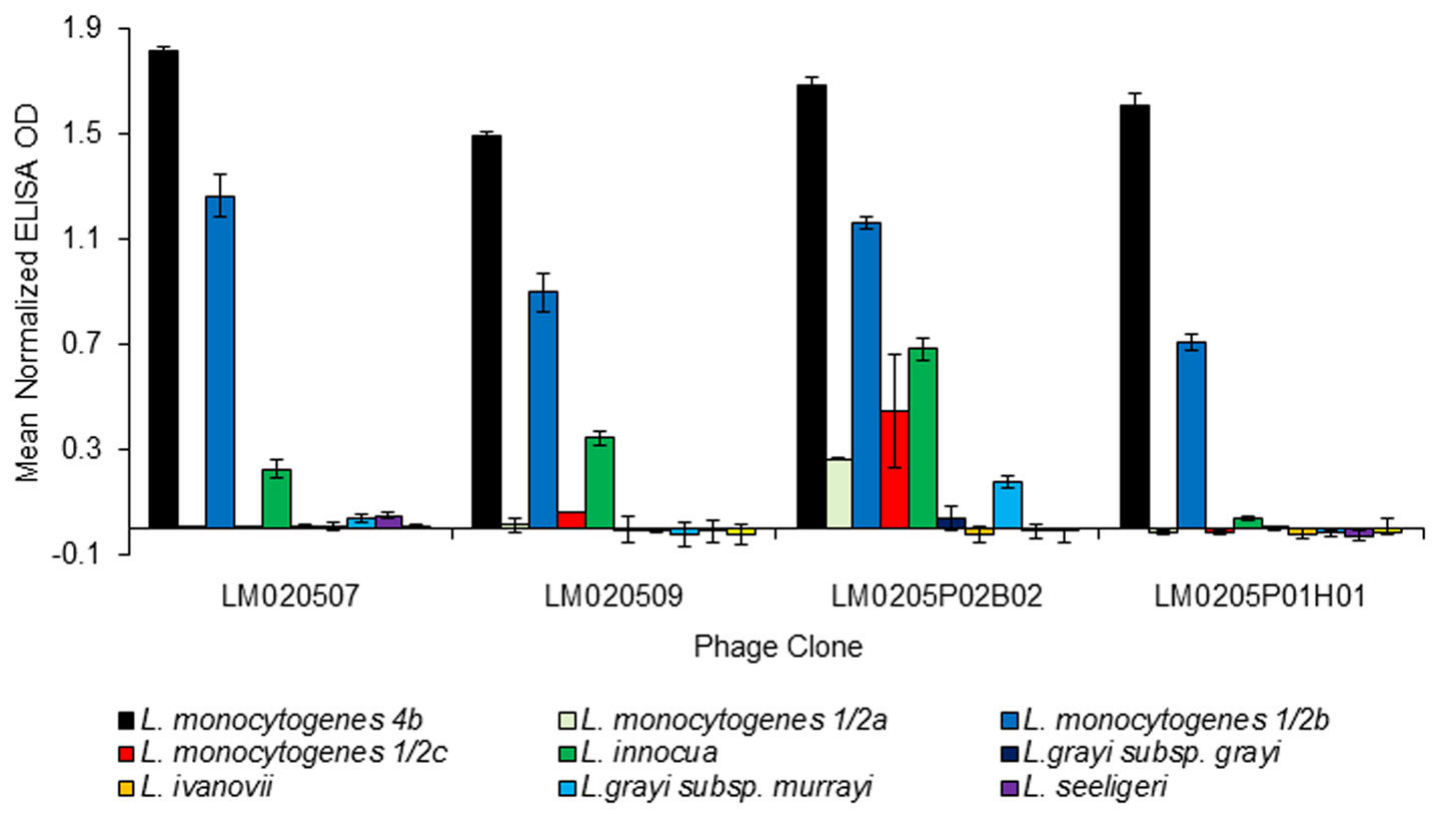
Phage binding ELISA specificity study of the four best *L. monocytogenes* 4b binding phage clones. Normalized data equates to relative OD signal after subtraction of background OD signal. Experiment performed in duplicate (n=2). Error bars represent ± standard deviation of mean.

Relative binding to *L. monocytogenes* 4b and *L. innocua* for seventeen selected phage clones demonstrated that none of the phage clones from the surface biopannings (rounds 3 and 4A) had higher binding to *L. monocytogenes* 4b than to *L. innocua*, whereas the twelve phage clones from solution biopanning round 5 did (Figure 3). On further evaluation of the four best *L. monocytogenes* 4b-binding phage clones, LM020507, LM020509, LM0205P02B02 and LM0205P01H01, with four *L. monocytogenes* serovars (4b, 1/2a, 1/2b and 1/2c) and six other *Listeria* spp., it was found that all four phage clones demonstrated higher binding to *L. monocytogenes* 4b than to the other three *L. monocytogenes* serovars (1/2a, 1/2b and 1/2c, Figure 4). All four phage clones exhibited binding to 1/2b and three of the four phage clones (exception of phage clone LM0205P02B02) exhibited no binding to *L. monocytogenes* 1/2a and 1/2c. The four phage clones exhibited minimal or no binding to *L**. grayi* subsp. *grayi**, **L**. ivanovii**, **L**. grayi* subsp. *murrayi**, **L**. seeligeri* and *L**. welshimeri*. While varying degrees of binding to *L. innocua* occurred, the fold difference results demonstrated that phage clones LM020507, LM020509 and LM0205P02B02 to have at least 2.5 times more binding capability to *L. monocytogenes* 4b than to *L. innocua* (8.0, 4.4 and 2.5 fold difference, respectively, data not shown). In fact, phage clone LM0205P01H01 demonstrated 43 times more binding capability to *L. monocytogenes* 4b than to *L. innocua* (data not shown). The results demonstrate the four phage clones are *L. monocytogenes*-specific.

The detection sensitivity of the four phage clones with *L. monocytogenes* 4b was examined using two phage clone concentrations (1x10^11^ and 1x10^12^ pfu ml^-1^). The LODs were 1x10^7^ pfu ml^-1^ for each phage clone regardless of the phage concentration (data not shown).

## Discussion

The objectives of this study were to produce phage display-derived peptide binders with the ability to distinguish pathogenic *L. monocytogenes* from other *Listeria* spp. and to evaluate these novel binders in a number of testing formats which would facilitate rapid detection of *L. monocytogenes*. A series of both surface and solution based biopannings were required to achieve the desired binding specificity for *L. monocytogenes*. The first three biopannings were surface based without subtraction against *L. innocua*, therefore it was not surprising that the one phage clone with higher specificity to *L. monocytogenes* 4b than to the other non-*Listeria* bacteria also demonstrated equally high binding to *L. innocua* (Figure 2A). *L. innocua* is the *Listeria* sp. genetically closest to *L. monocytogenes* [26], hence we hypothesised that subtraction against non-target *L. innocua* was a necessity to select for peptides with higher specificity to *L. monocytogenes*. The effect of subtraction in surface biopanning round 4A was exemplified by all tested phage clones exhibiting higher binding to *L. monocytogenes* 4b in the phage binding ELISA than to the other bacteria, including *L. innocua*. However, when the peptides expressed by these phage clones were chemically synthesised and evaluated (Table 3) they did not demonstrate higher specificity to *L. monocytogenes* 4b by MS + plate counts (exhibited no binding to *L. monocytogenes* 4b or *L. innocua*) or by sandwich ELISA (exhibited similar binding to *L. monocytogenes* 4b and *L. innocua*). The lack of *Listeria* cell capture by MS, which was observed with all peptide-coated beads tested during this study, was a surprising result given that we have recently demonstrated the applicability of phage display-derived peptides for MS of *Salmonella* spp., with equivalent or better capture capability observed with peptide-coated beads than for antibody-coated anti-*Salmonella* Dynabeads^®^ [33]. The commercially available antibody coated anti-*Listeria* Dynabeads® were able to capture both *L. monocytogenes* 4b and *L. innocua*, but were not able to differentiate between the two species. Paoli et al. [34] have previously described an *L. monocytogenes*-specific antibody fragment-coated bead but, to our knowledge, no publication exists describing an *L. monocytogenes*-specific peptide-coated bead, perhaps suggesting that the generation of such a bead is a difficult task. Paoli et al. [5] described *L. monocytogenes* as lacking surface epitopes which are both unique and antigenic, or having such epitopes but not effectively processing or presenting these during *in vivo* antibody production and maturation. Alternatively, the immunodominant epitopes may be shared between *Listeria* *species* making it difficult to identify the less immunogenic but crucially specific epitopes. This may explain why no *L. monocytogenes*-specific antibody coated magnetic beads are commercially available. However, in *vitro* selection by phage display biopanning does not rely on antigen immunogenicity, the lack of which is a major limitation of *in vivo* antibody production [35]. Furthermore, the biopanning incubation times are short (~ 1 hour) unlike during *in vivo* antibody production and maturation processes. Therefore, it is plausible that a window of opportunity may exist for phage display biopanning to successfully generate a *L. monocytogenes*-specific binder. It may be the case that the peptides used to coat magnetic beads during this study were true binders however, presentation of the *L. monocytogenes* epitopes during MS was not appropriate for binding, unlike during the biopanning process when the target was in suspension rather than bead-surface attached. Future studies could evaluate different MS conditions such as mixing speed or capture time.

The biopanning regime adopted during this study included two rounds of solution biopanning, with inclusion of subtraction biopanning, a similar approach to that employed by Paoli et al. [5], to determine if binding between peptides and target in a 3-dimensional environment (solution panning) as opposed to a 2-dimensional environment (surface biopanning) would result in binders with the desired specificity. The specificity study of twelve phage clones from solution biopanning round 5 demonstrated that all phage clones exhibited higher binding to *L. monocytogenes* 4b than *L. innocua* (Figure 3). In addition the results from the further evaluation study confirmed the four phage clones, LM020507, LM020509 LM0205P02B02 and LM0205P01H01, are *L. monocytogenes*-specific (Figure 4) with all four phage clones exhibiting binding to *L. monocytogenes* 4b and 1/2b and minimal or no binding to *L**. grayi* subsp. *grayi**, **L**. ivanovii**, **L**. grayi* subsp. *murrayi**, **L**. seeligeri* and *L**. welshimeri*, and *L. monocytogenes* 1/2a and 1/2c (with the exception of phage clone LM0205P02B02 which did exhibit binding to these two serovars). While all four phage clones exhibited varying degrees of binding to *L. innocua*, the fold difference results demonstrated the phage clones to have 2.5-43.5 times more specificity for *L. monocytogenes* than for *L. innocua*. Phage clone binding to *L. monocytogenes* 4b and 1/2b can be explained by the classification of these two serovars in the same phylogenetic division. The *L. monocytogenes* species has two major phylogenetic divisions as identified by numerous molecular subtyping techniques; division I consists of serovars 1/2b, 3b, 4b, 4d, and 4e, and division II consists of serovars 1/2a, 1/2c, 3a, and 3c [2]. The results suggest the four phage clones bind to the somatic (O) antigen, a serovar-specific protein, on the *L. monocytogenes* cell wall, explaining their binding to *L. monocytogenes* serovars and their lack of binding to other *Listeria* spp. Phage clone binding to *L. innocua* even after the inclusion of subtraction biopanning against *L. innocua* can be explained by a similar somatic (O) antigen, or other *L. monocytogenes* 4b-like surface antigen, between the two species. As mentioned earlier, Lan et al. [26] described *L. innocua* as being the species genetically closest to *L. monocytogenes* and reported that some *L. innocua* strains express *L. monocytogenes* 4b-like surface antigens. Overall, the results suggest that the inclusion of solution biopanning, and a possible synergistic effect of combined subtractive and solution biopanning, resulted in phages clones of the desired specificity for *L. monocytogenes*.

It would appear that solution biopanning also highlighted the amino acids most important in binding to *L. monocytogenes*. Many of the same amino acid residues were present (Table 4 and Tables S1 and S2). The twelve phage clones from round 5 with high binding capability for *L. monocytogenes* expressed peptide sequences with an N-terminus glycine residue, with six having proline as the adjacent amino acid residue (Table 4). The four phage clones with the highest specificity to *L. monocytogenes*, expressing peptide sequences GVIYDKPA-KLH, GPLATLHLPHKT, GPIRDIGPVMDH and GRIADLPPLKPN contain proline with increased frequency (frequency of 1, 2, 2 and 3 proline residues, respectively). According to Kay et al. [36] among the primary structures of many ligands for protein–protein interactions proline is critical, and the authors have identified protein-interaction domains that prefer proline-rich ligand sequences. In addition, these four phage clones were not obtained in the earlier surface biopanning rounds. In fact, the last clone exhibited 43 times more specificity for *L. monocytogenes* than for *L. innocua* and other *Listeria* spp. and was deduced by large scale DNA sequencing only. The authors believe incorporation of such high throughput capabilities for screening of phage clones, for both their DNA sequencing and binding capacities, would greatly increase the success rate of obtaining phage clone(s) with the desired target specificity. The use of a high throughout technique such as a microarray system could lead to better and faster screening, as has been demonstrated recently for the high throughput screening of hybridmonas [37].

In conclusion, the adopted biopanning regime, utilizing both surface and solution biopanning, resulted in an increase in both phage-target binding and consensus which are the desired outcomes of phage display biopanning, the purpose of which is to generate a unique library of high affinity phage-target binders which possess the amino acid residues most important for target binding. This proof-of-principle study successfully generated phage display-derived binders with the ability to distinguish *L. monocytogenes* from other *Listeria* spp. These binders have the potential to be used for the development of more rapid and specific detection methods for *L. monocytogenes* in food and clinical samples.

## Supporting Information

Table S1 Results from large scale DNA sequencing of 192 phage clones randomly selected after round 4B biopanning.
(DOCX)

Table S2 Results from large scale DNA sequencing of 192 phage clones randomly selected after round 5 biopanning.
(DOCX)

Table S3 Comparison of the frequency of occurrence of different peptide sequences expressed by phage clones randomly selected after round 4B and round 5 biopanning.
(DOCX)

Figure S1 Multiple sequence alignment for DNA sequences from alternative round 4B generated using ClustalW2 2.1 (http://www.ebi.ac.uk/Tools/msa/clustalw2/).
(DOCX)

Figure S2 Multiple sequence alignment for DNA sequences from round 5 generated using ClustalW2 2.1 (http://www.ebi.ac.uk/Tools/msa/clustalw2/).
(DOCX)
