# Supplementary material for: Phage Display-Derived Binders Able to Distinguish Listeria monocytogenes from Other Listeria Species
Source: PLoS One. 2013 Sep 10;8(9):e74312. doi: 10.1371/journal.pone.0074312 (PMC3769378; doi:10.1371/journal.pone.0074312)
Supplement: Table S2 — Results from large scale DNA sequencing of 192 phage clones randomly selected after round 5 biopanning. (DOCX) [file pone.0074312.s002.docx]

**Table S2** Results from large scale DNA sequencing of 192 phage clones randomly selected after round 5 biopanning.

| **Phage clone identifier** | **Peptide sequence** | **Frequency** |
| --- | --- | --- |
| >LM0205P01G07 | ANAGPIMTTSLL | 1 |
| >LM0205P02F08 | G*V*KPPQNVNR | 1 |
| >LM0205P02F02 | GAKHPAQPHMMY | 1 |
| >LM0205P01D05 | GAMHLPWHMGTL | 3 |
| >LM0205P01C08 | GAMHLSVAYGYA | 1 |
| >LM0205P01F08 | GEVDFNPR*DCA | 1 |
| >LM0205P01D03 | GIIYDNPRKELN | 1 |
| >LM0205P01A11 | GIIYSRGPEKLL | 1 |
| >LM0205P02A07 | GIIYTLPAARYD | 1 |
| >LM0205P02D05 | GIVFTLPALAHN | 2 |
| >LM0205P01A08 | GKLFSSLDGL*F | 1 |
| >LM0205P01A06 | GKLFSSPMDYDS | 4 |
| >LM0205P01A03 | GKLYSHPLNNAK | 5 |
| >LM0205P01A05 | GLLWTHPQTHGR | 18 |
| >LM0205P01G03 | GMIWNEPKTWPG | 1 |
| >LM0205P02B05 | GMIYVKPARPML | 1 |
| >LM0205P01B07 | GNLFASPQKMHR | 12 |
| >LM0205P01A07 | GPIFSAPT*TTI | 1 |
| >LM0205P01C04 | GPIFSNSWGLIT | 2 |
| >LM0205P01B06 | GPIFVNSDKGER | 1 |
| >LM0205P02D11 | GPIHVAAFKNMT | 1 |
| >LM0205P02B10 | GPILDMGFFNRE | 1 |
| >LM0205P02E10 | GPIMSLPHRTVG | 1 |
| >LM0205P01D08 | GPIMSLPTPTNL | 1 |
| >LM0205P02C09 | GPINSKPSHMHI | 1 |
| >LM0205P02B01 | GPIRDIGPVMDH | 3 |
| >LM0205P02C03 | GPIVDSGGTHPR | 1 |
| >LM0205P01H12 | GPIVSMPMPRLL | 1 |
| >LM0205P02H09 | GPIWDNMPSRQV | 1 |
| >LM0205P02B07 | GPIWSGRLIAQD | 1 |
| >LM0205P01D06 | GPIYETIKTRTP | 1 |
| >LM0205P02E01 | GPIYQQQNTILR | 1 |
| >LM0205P01C09 | GPIYSTQHMKTS | 3 |
| >LM0205P01A09 | GPLFDQGTQAYA | 2 |
| >LM0205P02C06 | GPLHSSPLKISS | 2 |
| >LM0205P01B09 | GPLISTPRHMNI | 4 |
| >LM0205P01B03 | GPLVDLGPGDLR | 6 |
| >LM0205P01G09 | GPLWTGQSQGSP | 1 |
| >LM0205P01B02 | GPLYESRMPQNH | 2 |
| >LM0205P01C11 | GPLYISSLTQLA | 1 |
| >LM0205P01H10 | GPLYIVSHDTPR | 1 |
| >LM0205P01G02 | GPVHSHPNDYSR | 2 |
| >LM0205P01C03 | GQVYDVPYSRPK | 2 |
| >LM0205P01H01 | GRIADLPPLKPN | 3 |
| >LM0205P01B10 | GRIATLPDPTPR | 2 |
| >LM0205P02G09 | GTDLD*AAAS*A | 1 |
| >LM0205P01E12 | GTIFDYGPHGYA | 1 |
| >LM0205P01A04 | GTIFDYGPPDMP | 13 |
| >LM0205P01A02 | GTIWSQPGAISL | 1 |
| >LM0205P01F03 | GVIWSDPKTASS | 1 |
| >LM0205P01A12 | GVIYDKPA*KLH | 11 |
| >LM0205P02E07 | GVIYDSHGPGRY | 1 |
| >LM0205P01D07 | GVIYSKPNSVQL | 7 |
| >LM0205P01G01 | GVIYSSDRDWRS | 1 |
| >LM0205P01C10 | GVIYTDSLTRPH | 1 |
| >LM0205P02A09 | GVMCKHPQTHGH | 1 |
| >LM0205P01B08 | KLHISKDHIYPT | 6 |
| >LM0205P01C02 | KQATFDDYPVAH | 5 |
| >LM0205P01E09 | LYAKKPLLNPNR | 2 |
| >LM0205P01E03 | NRPDSAQFWLHH | 4 |
| >LM0205P02E02 | QSWPAAA*AFTS | 1 |
| >LM0205P01D02 | TSMDSVSVIDLG | 1 |
| >LM0205P01H11 | TSSQGDRLYVYK | 2 |
| >LM0205P01E10 | TSWPSLSTSARS | 1 |
| >LM0205P01B11 | VNLEHGYYHAPS | 1 |
