# Supplementary material for: Phage Display-Derived Binders Able to Distinguish Listeria monocytogenes from Other Listeria Species
Source: PLoS One. 2013 Sep 10;8(9):e74312. doi: 10.1371/journal.pone.0074312 (PMC3769378; doi:10.1371/journal.pone.0074312)
Supplement: Table S3 — Comparison of the frequency of occurrence of different peptide sequences expressed by phage clones randomly selected after round 4B and round 5 biopanning. (DOCX) [file pone.0074312.s003.docx]

**Table S3** Comparison of the frequency of occurrence of different peptide sequences expressed by phage clones randomly selected after round 4B and round 5 biopanning.

| **Phage clone identifier** | **Peptide sequence** | **Frequency amongst round 4B**  **phage clones** | **Frequency amongst round 5 phage clones** |
| --- | --- | --- | --- |
| >LM0204P02E08 | ANATFHGYPTRS | 1 | 0 |
| >LM0204P01C07 | FFPREYYSIEAP | 1 | 0 |
| >LM0204P01A05 | GGNGPKGNNVVH | 1 | 0 |
| >LM0204P02B06 | GKIWTEPPPPKP | 1 | 0 |
| >LM0204P02G03 | GKIYNDPRMMSN | 1 | 0 |
| >LM0204P02F07 | GKLFSSPMDYDS | 1 | 4 |
| >LM0204P01G01 | GKLYSHPLNNAK | 2 | 5 |
| >LM0204P02C07 | GKYHEKHNENMH | 1 | 0 |
| >LM0204P01H06 | GLIWDLSWCSSK | 1 | 0 |
| >LM0204P01G06 | GLLWTHPQTHGR | 1 | 18 |
| >LM0204P02A04 | GMIWNEPKTWPG | 1 | 1 |
| >LM0204P01D01 | GMIWSRPSAEKF | 1 | 0 |
| >LM0204P01H10 | GNLFASPQKMYR | 2 | 0 |
| >LM0204P01F04 | GPIATLPKGGGQ | 1 | 0 |
| >LM0204P01C02 | GPIFSNSWGLIT | 5 | 2 |
| >LM0204P01F09 | GPIHEAPSSPSG | 2 | 0 |
| >LM0204P01C10 | GPIIA*TYPKRE | 1 | 0 |
| >LM0204P01D07 | GPILDMGFFNRE | 1 | 1 |
| >LM0204P02C10 | GPIMSLPHRTVG | 1 | 1 |
| >LM0204P02E07 | GPIWSSIQTLPT | 1 | 0 |
| >LM0204P01H03 | GPIYSSVKDGQR | 1 | 0 |
| >LM0204P02C02 | GPIYSTQHMKTS | 1 | 3 |
| >LM0204P02G04 | GPIYTDKSELGN | 1 | 0 |
| >LM0204P02H04 | GPLATLHLPHKT | 1 | 0 |
| >LM0204P02H01 | GPLFDQGTQAYA | 1 | 2 |
| >LM0204P02E04 | GPLFITSAPPTK | 1 | 0 |
| >LM0204P01A01 | GPLFSDPEPAKN | 1 | 0 |
| >LM0204P02F09 | GPLISTPRHMNI | 1 | 4 |
| >LM0204P02G05 | GPLVDLGPGDLR | 1 | 6 |
| >LM0204P01B04 | GPLWTGQSQGSP | 3 | 1 |
| >LM0204P02B07 | GPLYESRMPQNH | 1 | 2 |
| >LM0204P02E05 | GPLYESSQVIRA | 1 | 0 |
| >LM0204P02F05 | GPLYSSMASALA | 1 | 0 |
| >LM0204P02A06 | GPLYSTNLPTRN | 2 | 0 |
| >LM0204P02H05 | GPLYSYPFSMIE | 1 | 0 |
| >LM0204P01D02 | GPSHNTLSPLLT | 1 | 0 |
| >LM0204P02D11 | GPVHSHPNDYSR | 1 | 2 |
| >LM0204P02B01 | GPVLDPLTPSTI | 1 | 0 |
| >LM0204P01B06 | GQIYTTRDSLLG | 1 | 0 |
| >LM0204P01D03 | GQKPT*NLDLKL | 1 | 0 |
| >LM0204P01H02 | GQVYDVPYSRPK | 2 | 2 |
| >LM0204P02C08 | GSIYAHPRWLKW | 1 | 0 |
| >LM0204P02G10 | GTIMTLANTERP | 1 | 0 |
| >LM0204P02D02 | GTIQVHPPAPAR | 1 | 0 |
| >LM0204P02C04 | GVIWSDPKTASS | 1 | 1 |
| >LM0204P02G09 | GVIYSKPNSVQL | 1 | 7 |
| >LM0204P01G03 | GVIYSSDRDWRS | 1 | 1 |
| >LM0204P02H08 | GVIYSTHDTRPY | 1 | 0 |
| >LM0204P02F02 | GVLHSSPNHRWQ | 1 | 0 |
| >LM0204P01G02 | GWHKHKSMSAPL | 1 | 0 |
| >LM0204P01H05 | HLINTNAQIAQR | 1 | 0 |
| >LM0204P02D05 | IQLEMGGTRFHR | 2 | 0 |
| >LM0204P02F04 | KDDASPAWNSRH | 1 | 0 |
| >LM0204P02D12 | KHMMINAYRMTE | 1 | 0 |
| >LM0204P01B05 | KIHKTESTPAWF | 1 | 0 |
| >LM0204P02H07 | KKGDV**L*LRR | 1 | 0 |
| >LM0204P01F01 | KLHISKDHIYPT | 4 | 6 |
| >LM0204P01D06 | KNLHVGSYPQPI | 1 | 0 |
| >LM0204P01H04 | KPHAHKHNDYFL | 1 | 0 |
| >LM0204P02E01 | KPHHPHKIPYTN | 1 | 0 |
| >LM0204P01D05 | KPHNMTELHHKH | 1 | 0 |
| >LM0204P02G01 | KPHYDHRLHQPI | 1 | 0 |
| >LM0204P01A06 | KQATFDDYPVAH | 24 | 5 |
| >LM0204P01F11 | KQSDVR*VSWWA | 1 | 0 |
| >LM0204P02A11 | KRRAKQKTGEQR | 1 | 0 |
| >LM0204P01B01 | LDLQTPGHKWSQ | 1 | 0 |
| >LM0204P01F05 | LLPPT*ATVGAR | 1 | 0 |
| >LM0204P02D04 | LSCTTSVACLQT | 1 | 0 |
| >LM0204P02G02 | NVATKSSGHNMR | 1 | 0 |
| >LM0204P01F10 | RQVRMHPLDSWS | 1 | 0 |
| >LM0204P01E03 | RRKMKQTEKMKI | 1 | 0 |
| >LM0204P01C01 | SKSRAKGKQAKN | 1 | 0 |
| >LM0204P01E04 | SLNRKKRRTHAK | 1 | 0 |
| >LM0204P02F06 | SLRQVNTHTWLT | 1 | 0 |
| >LM0204P01G04 | TDAKMRAKFPGH | 1 | 0 |
| >LM0204P01F12 | TQARCNEYPVGH | 1 | 0 |
| >LM0204P02F03 | TSMDSVSVIDLG | 1 | 1 |
| >LM0204P01A03 | VNLEHGYYHAPS | 3 | 1 |
| >LM0204P01A04 | VNLQTGWYTMAS | 7 | 0 |
| >LM0204P02F10 | VNSADWVVCDGV | 1 | 0 |
| >LM0204P01G05 | VSLPMGFYSMNS | 1 | 0 |
| >LM0204P02A03 | YLPISQTHNRNV | 1 | 0 |
