# Supplementary material for: Phage Display-Derived Binders Able to Distinguish Listeria monocytogenes from Other Listeria Species
Source: PLoS One. 2013 Sep 10;8(9):e74312. doi: 10.1371/journal.pone.0074312 (PMC3769378; doi:10.1371/journal.pone.0074312)
Supplement: Figure S1 — Multiple sequence alignment for DNA sequences from alternative round 4B generated using ClustalW2 2.1 (http://www.ebi.ac.uk/Tools/msa/clustalw2/). (DOCX) [file pone.0074312.s004.docx]

**Figure S1** Multiple sequence alignment for DNA sequences from alternative round 4B generated using ClustalW2 2.1 (<http://www.ebi.ac.uk/Tools/msa/clustalw2/>)

LM0204P02A11 ---KRRAKQKTGEQR-- 12

LM0204P01C01 --SKSRAKGKQAKN--- 12

LM0204P01G04 --TDAKMRAKFPGH--- 12

LM0204P02F04 ---KDDASPAWNSRH-- 12

LM0204P01B05 KIHKTESTPAWF----- 12

LM0204P01F01 KLHISKDH-IYPT---- 12

LM0204P01H04 KPHAHKHN-DYFL---- 12

LM0204P01A05 --GGNGPKGNNVVH--- 12

LM0204P02C07 --GKYHEKHNENMH--- 12

LM0204P01D05 --KPHNMTELHHKH--- 12

LM0204P02G01 --KPHYDHRLHQPI--- 12

LM0204P02B06 --GKIWTEPPPPKP--- 12

LM0204P02D02 --GTIQVHPPAPAR--- 12

LM0204P02A04 --GMIWNEPKTWPG--- 12

LM0204P02C04 --GVIWSDPKTASS--- 12

LM0204P01D01 --GMIWSRPSAEKF--- 12

LM0204P02G03 --GKIYNDPRMMSN--- 12

LM0204P02C08 --GSIYAHPRWLKW--- 12

LM0204P01F09 --GPIHEAPSSPSG--- 12

LM0204P02C10 --GPIMSLPHRTVG--- 12

LM0204P02E07 --GPIWSSIQTLPT--- 12

LM0204P02H08 --GVIYSTHDTRPY--- 12

LM0204P01H03 --GPIYSSVKDGQR--- 12

LM0204P01G03 --GVIYSSDRDWRS--- 12

LM0204P02C02 --GPIYSTQHMKTS--- 12

LM0204P02G04 --GPIYTDK-SELGN-- 12

LM0204P01B06 --GQIYTTRDSLLG--- 12

LM0204P01D02 --GPSHNTLSPLLT--- 12

LM0204P01H06 --GLIWDLSWCSSK--- 12

LM0204P01C02 --GPIFSNSWGLIT--- 12

LM0204P01C10 --GPIIA-TYPKRE--- 11

LM0204P01D07 --GPILDMGFFNRE--- 12

LM0204P01F04 --GPIATLPKGGGQ--- 12

LM0204P02H04 --GPLATLHLPHKT--- 12

LM0204P02H01 --GPLFDQGTQAYA--- 12

LM0204P02G05 --GPLVDLGPGDLR--- 12

LM0204P01A01 --GPLFSDPEPAKN--- 12

LM0204P02A06 --GPLYSTNLPTRN--- 12

LM0204P02E04 --GPLFITSAPPTK--- 12

LM0204P02B07 --GPLYESRMPQNH--- 12

LM0204P02E05 --GPLYESSQVIRA--- 12

LM0204P02F05 --GPLYSSMASALA--- 12

LM0204P01G06 --GLLWTHPQTHGR--- 12

LM0204P01B04 --GPLWTG-QSQGSP-- 12

LM0204P01H05 --HLINTN-AQIAQR-- 12

LM0204P01F05 ---LLPPT-ATVGAR-- 11

LM0204P02F07 --GKLFSSPMDYDS--- 12

LM0204P01H10 --GNLFASPQKMYR--- 12

LM0204P02F09 --GPLISTPRHMNI--- 12

LM0204P02H05 --GPLYSYPFSMIE--- 12

LM0204P01G01 --GKLYSHPLNNAK--- 12

LM0204P02G09 --GVIYSKPNSVQL--- 12

LM0204P02F02 --GVLHSSPNHRWQ--- 12

LM0204P02D11 --GPVHSHP-NDYSR-- 12

LM0204P01F10 --RQVRMHPLDSWS--- 12

LM0204P02B01 --GPVLDPLTPSTI--- 12

LM0204P01B01 -----LDLQTPGHKWSQ 12

LM0204P02H07 -----KKGDV--L-LRR 9

LM0204P01F11 -----KQSDVR-VSWWA 11

LM0204P01A06 --KQATFDDYPVAH--- 12

LM0204P01F12 --TQARCNEYPVGH--- 12

LM0204P02E08 --ANATFHGYPTRS--- 12

LM0204P01A03 --VNLEHGYYHAPS--- 12

LM0204P01A04 --VNLQTGWYTMAS--- 12

LM0204P01G05 --VSLPMGFYSMNS--- 12

LM0204P01D06 --KNLHVGSYPQPI--- 12

LM0204P02G10 --GTIMTLANTERP--- 12

LM0204P02E01 --KPHHPHKIPYTN--- 12

LM0204P02A03 -YLPISQTHNRNV---- 12

LM0204P01H02 --GQVYDVPYSRPK--- 12

LM0204P01E04 --SLNRKKRRTHAK--- 12

LM0204P01D03 --GQKPT-NLDLKL--- 11

LM0204P01E03 --RRKMKQTEKMKI--- 12

LM0204P02D04 --LSCTTSVACLQT--- 12

LM0204P02G02 --NVATKSSGHNMR--- 12

LM0204P02D05 --IQLEMGGTRFHR--- 12

LM0204P02F06 --SLRQVNTHTWLT--- 12

LM0204P02F10 --VNSADWVVCDGV--- 12

LM0204P02F03 --TSMDSVSVIDLG--- 12

LM0204P01G02 --GWHKHKSMSAPL--- 12

LM0204P02D12 --KHMMINAYRMTE--- 12

LM0204P01C07 ---FFPREYYSIEAP-- 12
