# Supplementary material for: Phage Display-Derived Binders Able to Distinguish Listeria monocytogenes from Other Listeria Species
Source: PLoS One. 2013 Sep 10;8(9):e74312. doi: 10.1371/journal.pone.0074312 (PMC3769378; doi:10.1371/journal.pone.0074312)
Supplement: Figure S2 — Multiple sequence alignment for DNA sequences from round 5 generated using ClustalW2 2.1 (http://www.ebi.ac.uk/Tools/msa/clustalw2/). (DOCX) [file pone.0074312.s005.docx]

**Figure S2** Multiple sequence alignment for DNA sequences from round 5 generated using ClustalW2 2.1 (<http://www.ebi.ac.uk/Tools/msa/clustalw2/>)

LM0205P01D05 ---GAMHLPWH-MGTL- 12

LM0205P01C08 ---GAMHLSVA-YGYA- 12

LM0205P01G07 ANAGPIMTTSL-L---- 12

LM0205P01C10 ---GVIYTDSL-TRPH- 12

LM0205P01B02 ---GPLYESRM-PQNH- 12

LM0205P01C11 ---GPLYISSL-TQLA- 12

LM0205P01H10 ---GPLYIVSH-DTPR- 12

LM0205P01D06 ---GPIYETIK-TRTP- 12

LM0205P01C09 ---GPIYSTQH-MKTS- 12

LM0205P02E01 ---GPIYQQQN-TILR- 12

LM0205P02B07 ---GPIWSGRL-IAQD- 12

LM0205P01G09 ---GPLWTGQS-QGSP- 12

LM0205P01C04 ---GPIFSNS--WGLIT 12

LM0205P01B06 ---GPIFVNSD-KGER- 12

LM0205P02D11 ---GPIHVAAF-KNMT- 12

LM0205P02B10 ---GPILDMGF-FNRE- 12

LM0205P02B01 ---GPIRDIGP-VMDH- 12

LM0205P02H09 ---GPIWDNMP-SRQV- 12

LM0205P02C03 ---GPIVDSGG-THPR- 12

LM0205P01B03 ---GPLVDLGP-GDLR- 12

LM0205P01E12 ---GTIFDYGP-HGYA- 12

LM0205P01A04 ---GTIFDYGP-PDMP- 12

LM0205P01A09 ---GPLFDQGT-QAYA- 12

LM0205P01D03 ---GIIYD-NP-RKELN 12

LM0205P01A11 ---GIIYSRGP-EKLL- 12

LM0205P02B05 ---GMIYVKPA-RPML- 12

LM0205P01A12 ---GVIYDKPA--KLH- 11

LM0205P01D07 ---GVIYSKPN-SVQL- 12

LM0205P02E07 ---GVIYDSHG-PGRY- 12

LM0205P01G01 ---GVIYSSDR-DWRS- 12

LM0205P01G03 ---GMIWNEPK-TWPG- 12

LM0205P01F03 ---GVIWSDPK-TASS- 12

LM0205P01A02 ---GTIWSQPG-AISL- 12

LM0205P01A08 ---GKLFSS-L-DGL-F 11

LM0205P01A06 ---GKLFSSPM-DYDS- 12

LM0205P01B07 ---GNLFASPQ-KMHR- 12

LM0205P02C06 ---GPLHSSPL-KISS- 12

LM0205P01A03 ---GKLYSHPL-NNAK- 12

LM0205P01G02 ---GPVHSHPN-DYSR- 12

LM0205P02E10 ---GPIMSLPH-RTVG- 12

LM0205P01D08 ---GPIMSLPT-PTNL- 12

LM0205P01A07 ---GPIFSAPT--TTI- 11

LM0205P01H12 ---GPIVSMPM-PRLL- 12

LM0205P02C09 ---GPINSKPS-HMHI- 12

LM0205P01B09 ---GPLISTPR-HMNI- 12

LM0205P02A07 ---GIIYTLPA-ARYD- 12

LM0205P02D05 ---GIVFTLPA-LAHN- 12

LM0205P01H01 ---GRIADLPP-LKPN- 12

LM0205P01B10 ---GRIATLPD-PTPR- 12

LM0205P01C03 ---GQVYDVPY-SRPK- 12

LM0205P02F08 ---G-V-KPPQ-NVNR- 10

LM0205P01E09 ---LYAKKPLL-NPNR- 12

LM0205P01A05 ---GLLWTHPQ-THGR- 12

LM0205P02A09 ---GVMCKHPQ-THGH- 12

LM0205P01D02 ---TSMDSVSV-IDLG- 12

LM0205P01E10 ---TSWPSLST-SARS- 12

LM0205P02F02 ---GAKHPAQP-HMMY- 12

LM0205P02G09 ---GTDLD-AA-AS-A- 10

LM0205P02E02 ----QSWPAAA-AFTS- 11

LM0205P01F08 ---GEVDFNPR-DCA-- 11

LM0205P01E03 ---NRPDSAQFWLHH-- 12

LM0205P01B11 ----VNLEHGYYHAPS- 12

LM0205P01C02 --KQATFDDYP-VAH-- 12

LM0205P01B08 ----KLHISKDHIYPT- 12

LM0205P01H11 ---TSSQGDRL-YVYK- 12
